# A Dual Targeting Magnetic Nanoparticle for Human Cancer Detection

**DOI:** 10.1186/s11671-019-3049-0

**Published:** 2019-07-09

**Authors:** Siwen Wu, Xiyu Liu, Jian He, Huiling Wang, Yiqun Luo, Wenlin Gong, Yanmei Li, Yong Huang, Liping Zhong, Yongxiang Zhao

**Affiliations:** 1 National Center for International Research of Bio-targeting Theranostics, Guangxi Key Laboratory of Bio-targeting Theranostics, Collaborative InnovationCenter for Targeting Tumor Diagnosis and Therapy, Guangxi Medical University, Nanning, 530021 Guangxi China; 2 School of Preclinical Medicine, Guangxi Medical University, Nanning, 530021 Guangxi China

**Keywords:** Lymphatic endothelial cells, Podoplanin, LYVE-1, Magnetic nanoparticles

## Abstract

Malignant tumors are a major threat to human life and high lymphatic vessel density is often associated with metastatic tumors. With the discovery of molecules targeted at the lymphatic system such as lymphatic vessel endothelial hyaluronan receptor 1 (LYVE-1) and Podoplanin, many studies have been performed to determine the role of lymphatic endothelial cells (LECs) in tumor metastasis. However, disadvantages such as non-specificity and high cost limit their research and diagnostic applications. In this study, Fe_3_O_4_@KCTS, a core-shell type of magnetic nanoparticles, was prepared by activating Fe_3_O_4_ with carbodiimide and cross-linking it with α-ketoglutarate chitosan (KCTS). The LYVE-1 and Podoplanin antibodies were then incorporated onto the surface of these magnetic nanoparticles and as a result, dual-targeting magnetic nanoprobes were developed. The experimental tests of this study demonstrated that a dual-targeting magnetic nanoprobe with high-purity LECs from tumor tissues was successfully developed, providing a basis for clinical application of LECs in colorectal cancer treatment as well as in early clinical diagnosis using bimodal imaging.

## Introduction

Malignant tumors pose a great threat to human health [1]. Previous studies have shown that the tumor microenvironment affects the biological characteristics of tumors, such as tumor-related metastases [2, 3]. However, the potential mechanisms, particularly the process by which lymphatic endothelial cells (LECs) promote tumor metastasis, are still not clear [4]. It is therefore necessary to design diagnostic strategies for tumors based on targeting the lymphatic vessels. The lack of lymphatic-specific markers, especially those that distinguish LECs from vascular endothelium, limits research on LECs functions. In the recent years, LECs specific markers, such as lymphatic vessel endothelial hyaluronan receptor 1 (LYVE-1) [5, 6], Podoplanin [7], vascular endothelial growth factor receptor 3 (VEGFR-3) [8], and Prox-1 have been identified [9]. This has accelerated research on tumor lymphatic vessels, especially by targeting tumor endolymphatic vessels to diagnose tumors. It has also enabled studies on the mechanism of tumor metastasis by sorting of tumor endolymphatic endothelial cells using immunomagnetic beads [10]. However, similar to many other markers used in molecular pathology, none of the LECs-associated molecular markers are completely specific for LECs. Due to the transient expression of Prox-1 in the nucleus [11], it is not suitable for clinical applications. Although VEGFR-3 is expressed in LECs, it lacks lymphatic specificity in cancer because it is also expressed in vascular epithelium [8]. LYVE-1 is specifically expressed in LECs as well as in normal hepatic sinusoids, spleen endothelium, high endothelial venules, as well as activated tissue macrophages but it is not expressed in vascular epithelium [7]. Podoplanin is not expressed in vascular epithelial cells but it is a specific marker for LECs [7, 12].

Magnetic ferroferric oxide, nano gold, quantum dots, liposomes, and titanium oxide are widely used in cancer diagnosis and treatment because of their high biocompatibility, large specific surface area, and easy to modify with other biological functional molecules [13, 14]. In addition to the above characteristics, magnetic ferroferric oxide (Fe_3_O_4_) has a narrow size distribution, good dispersibility, high paramagnetism, and controllable size making it suitable for magnetic resonance imaging [15]. Among them, superparamagnetic iron oxide coated with antibodies has been widely used in cell separation, recognition, and early diagnosis of tumors [16, 17]. As a natural high molecular substance, chitosan surface is rich in hydroxyl and amino groups, and has been widely used due to its excellent properties such as biocompatibility, blood compatibility, safety, and microbial degradability [18]. Therefore, the combination of chitosan or chitosan derivative and magnetic Fe_3_O_4_ is more conducive for biological applications, because it can prevent magnetic beads from agglomerating with each other to form magnetic fluids. For this reason, it produces a better dispersion performance.

In tumor research, detection of lymphatic vessels in tumors is based on direct labeling with antibodies, or through an antibody combined with magnetic beads to sort lymphatic endothelial cells. Here, to target intratumoral lymphatic vessels more accurately, we designed a multi-targeted nanoprobe, i.e., two highly specific antibodies against lymphatic endothelial cells, anti-Lyve-1 antibody and anti-podplanin antibody. These antibodies were bound to chitosan-coated iron tetroxide nanoparticles. Compared with other nanoprobes, the nanoprobes designed in this study containing two antibodies are more accurate for lymphatic vessel detection and the isolated lymphatic endothelial cells are therefore more pure.

In this study, for rapid enrichment and isolation of LECs form human cancer cells, a ligand with a relatively high specificity for anti-LYVE-1 antibody and anti-podplanin antibody were used to facilitate binding to the chitosan-coated superparamagnetic nanoparticles to prepare magnetic beads for selecting LECs. It was also verified that the probe was capable of bimodal diagnosis of solid tumors.

## Materials and Method

### Cell Line and Cell Culture Conditions

The colon cancer cell line (HT29), which was purchased from the ATCC cell bank, was cultured at 37 °C in Dulbecco’s modified Eagle medium (DMEM) (Gibco, Grand Island, NY, USA) that contains 10% fetal bovine serum (Gibco, Grand Island, NY, USA), 100 U/mL penicillin, and 100 μg/mL streptomycin, under 5% CO_2_.

### Experimental Animal Model

Forty NOD/SCID female mice (4–6 weeks old) were purchased from Beijing Vital River Laboratory Animal Technology Company (Beijing, China). HT29 cells grown in log phase were washed three times with phosphate-buffered saline (PBS). After digesting them with trypsin, the cells were centrifuged at 1000 rpm for 5 min. The cells were then re-suspended in PBS and the cell concentration was adjusted to 2 × 10^7^/mL. Each mouse was injected with 200 μL of the cell suspension. All experimental protocols were approved by the Animal Ethics Committee of Guangxi Medical University (Guangxi, China).

### Preparation and Characterization of Fe_3_O_4_@KCTS-LECs-Double Antibody Magnetic Nanoprobes

Chitosan (Xi’an Ruixi Biotechnology) was carboxylated with α-ketoglutaric acid to obtain α-ketoglutarate carboxymethyl Chitosan (KCTS) rich in CO-groups on its surface [19]. Fe_3_O_4_ (Xi’an Ruixi Biotechnology) was used as the core and KCTS was set as the basic skeleton structure. After activating Fe_3_O_4_ nanoparticles with carbodiimide, Fe_3_O_4_@KCTS was formed by combining the covalent bond of KCTS-COOH and surface-OH. The selected LECs antibodies were specifically to human LYVE-1 APC-conjugated antibody (R&D Systems®, Cat. No. FAB20892A) and anti-podoplanin antibody (FITC) (Abcam, Cat. No. ab205333). These antibodies were cross-linked covalently to carboxylated Fe_3_O_4_@KCTS using activated EDC/NHS. Consequently, magnetic nanoprobes modified with LECs double antibodies, referred to Fe_3_O_4_@KCTS-LECs-Double antibody magnetic nanoprobe (0.1 mg/mL) were obtained. The double antibody-conjugated nanoparticles were preserved in darkness at 4 °C.

The morphology and size distribution of prepared Fe_3_O_4_@KCTS-LECs-Double antibody magnetic nanoparticles were evaluated using transmission electron microscope (TEM; H-7650, Japan). The particle size (Size), Zeta potential, and Particle Dispersion Index (PDI) of the Fe_3_O_4_@KCTS-Double antibody were measured using dynamic light scattering (DLS; PRO3000, Japan), and their photoluminescence was measured with a fluorescence spectrophotometer (FL-7000, Perkin Elmer, USA).

### Immunohistochemistry and Immunofluorescence

Frozen tissue sections were prepared and put in ice acetone for 10 min and then immersed in PBS solution for 10 min. After blocking in 1% BSA and washing three times with PBS, the sections were incubated with human LYVE-1 APC-conjugated antibody and anti-podoplanin antibody (FITC) at 4 °C for 30 min. After incubation, the sections were immersed three times in PBS solution after which a 4′,6-diamidino-2-phenylindole (DAPI) staining solution was added to the tumor tissue to completely cover the tissues. The sections were incubated again for 5 min at room temperature. Thereafter, they were washed three times with PBS, followed by addition of anti-fluorescence quenching agent when the sections were completely dry. Finally, the sections were mounted on a cover glass and observed under a confocal microscope (Nikon DS-Ri1; Nikon, Tokyo, Japan).

Colon cancer tissues preserved in formalin were paraffin-embedded for this study. Dewaxed sections of HT29 cancer xenografts were blocked with 3% hydrogen peroxide, blocked in 5% normal serum, and incubated with anti-LYVE-1 antibody (Abcam, ab10278, UK) overnight at 4 °C. Next, the biotin-labeled secondary antibody goat anti-rabbit IgG H&L (HRP) (ab205718) in the labeling reagent of horseradish peroxidase-conjugated superstreptavidin was incubated for 30 min. The color was measured using 3,3′-diaminobenzidine (DAB) solution.

### Cell Purification

The average volume of the tumors was about 1.5 × 1 × 1 cm^3^. Before removing the tumors, the mice were disinfected by immersing them in 75% ethanol for 3–5 min. The disinfected mice skin was cut with sterile scissors and sterile forceps on a clean bench. The tumor tissue was carefully excised and placed in a petri dish containing PBS. The soaked tumor tissue was then cut with ophthalmic scissors and placed in a sterile plate. The shredded tissue was pipetted into a 50 mL centrifuge tube using a pasteurized tube. Three times volume of collagenase I was then added into the tube, and vortexed for 10 min to obtain a collagenase I-tumor tissue mixture. The mixture was then placed in a 37 °C water bath for 20 min and this was repeated five times. At the end of digestion, collagenase I was stopped by adding a complete medium and the mixture was allowed to stand for 2 min. After centrifugation at 300 g for 10 min, the tumors were washed twice with PBS, then re-suspended in 10 mL PBS, and slowly filtered through a sieve with a pore size of 75 μm (200 mesh). The filtered cells were counted, averaged into two 15 mL centrifuge tubes, re-suspended in PBS, then centrifuged at 300 g for 10 min and then washed three times. (1) MACS magnetic bead sorting method: the washed cells were re-suspended in 100 μL buffer. A solution containing PBS pH 7.2, 0.5% BSA, and 2 mM EDTA was prepared by diluting MACS BSA stock solution (Cat. No. 130-091-376) to 1:20 with auto MACS™ rinsing solution (Cat. No. 130-091-222). Next, 10 μL human LYVE-1 APC-conjugated antibody was added and then incubated at 4 °C for 15 min in the dark. Finally, it was washed three times with 2 mL buffer solution. After centrifugation, 80 μL buffer solution was added to re-suspend the cells, and 20 μL of anti-APC fluorescein magnetic beads solution was added. The mixture was incubated for 15 min, washed three times, then centrifuged, and mixed well with 300 μL of buffer solution. Finally, the positive cells in the column were quickly knocked down with 3 mL buffer solution. The resulting cells were cultured in a six-well plate. (2) Fe_3_O_4_@KCTS-LECs-double antibody magnetic nanoparticle sorting method: the washed cells were re-suspended in 200 μL buffer, mixed with Fe_3_O_4_@KCTS-LECs-double antibody magnetic nanoparticles, incubated at 4 °C for 20 min in the dark, then washed three times with 4 mL buffer, centrifuged, and re-suspended in 160 μL buffer. Finally, the cells were eluted using a magnet and cultured in a 6-well plate.

### Immunofluorescence of Cells Sorted by Different Methods

The cells sorted by different sorting methods were adjusted to a cell concentration of 5 × 10^4^/mL with PBS, and evenly spread in a 12-well plate. One milliliter of the cell suspension was added to each well and then cultured at 37 °C for 24 h in 5% CO_2_ surrounding. The old medium was then discarded, and cells were washed three times with PBS solution. Then, 200 μL of 4% paraformaldehyde was added to each well, and the cells were kept at room temperature for 10 min. The cells were then washed three times with PBS solution, human LYVE-1 APC-conjugated antibody, and anti-podoplanin antibody (FITC) were added. After incubation in the dark at 4 °C for 2 h, the cells were washed three times. Further, 50 μL DAPI was added to each well and incubated at room temperature for 10 min to stain the nuclei blue followed by washing three times with PBS. Lastly, the anti-fluorescence quencher was dropped in the center of the slide, and the cell-covered side was placed on a glass slide and observed using a laser scanning confocal microscope.

### Flow Cytometry of Cells Sorted by Different Methods

The cells sorted by different methods were re-suspended with human LYVE-1 APC-conjugated antibody and anti-podoplanin antibody (FITC) antibody in 200 μL binding buffer (10 mM HEPES/NaOH pH 7.4, 140 mM NaCl, 2.5 mM CaCl), and incubated at 4 °C for 1 h in the dark, then washed three times with PBS. The cells were detected and analyzed with flow cytometry (BD Accury6; Becton Dickinson, San Jose, CA, USA) then analyzed by FlowJo software (Tree Star Inc., Ashland, OR, USA).

### Detection of LECs Activity

Logarithmic growth phase cells sorted by different methods were collected, digested, and counted. The cell concentration was adjusted to 2 × 10^5^ cells/mL and 50 μL of these cells was added into each well of a Matrigel-coated μ-slide plate and incubated in a 5% CO_2_ incubator at 37 °C for 6 h. The medium was separated and discarded with pasteurized dropper followed by addition of PBS solution containing 1 μg/mL calcein AM (Invitrogen, Cat. No. c3099). The cells were then incubated at room temperature for 1 h in the dark, washed with PBS, and photographed under a microscope (Nikon, Japan). Thereafter, 1 × 10^5^ cells were mixed with 500 μL complete medium containing 20 μg/mL of DiI-ac-LDL (Molecular Probes, Invitrogen), and then inoculated in a 24-well plate 37 °C for 4 h. The old culture solution was removed and the cells were washed three times in PBS. Then, 50 μL of DAPI solution was added to each well and incubated for 10 min at room temperature to stain the nuclei blue. After washing three times with PBS, they were finally imaged under a microscope.

### MR and Fluorescence Imaging In Vivo

The following steps were followed to determine whether the tumor was targeted by Fe_3_O_4_@KCTS-LECs-double antibody magnetic nanoparticles in animals. The NOD/SCID mouse subcutaneous xenograft model of colon cancer was used for magnetic resonance or fluorescence scanning. T2-weighted imaging was conducted in a specific condition that included field of view (80 × 80 mm), echo time (69 ms), and layer thickness (2.0 mm) using a 3.0T MRI scanner (Discovery 750, gE, germany), having a 40 mm diameter mouse volume coil. The NOD/SCID mice were divided into two groups (*n* = 3); the experimental group and the receptor blocking group. All mice were injected with Fe_3_O_4_@KCTS-LECs-double antibody (0.5 mmol/kg) magnetic nanoparticles via the tail vein. Each mouse was scanned at four specific time points: before injection, 0.5  h, 12 h, and 24 h after injection. In the receptor blocking group, each mouse was injected with Anti-LYVE-1 antibody (Abeam, ab10278, UK) and anti-podoplanin antibody (Abcam, ab10288, UK) before injection with Fe_3_O_4_@KCTS-LECs-double antibody. MRI scans were carried out at the same four time points. Fluorescence images were taken using the small animal in vivo imaging system (Bruker, USA). The time points for grouping, drug injection and scanning were as mentioned above.

### Toxicity of Fe_3_O_4_@KCTS-LECs-Double Antibody Magnetic Nanoprobe

Toxicity of Fe_3_O_4_@KCTS-LECs-double antibody magnetic nanoprobe against LECs was evaluated by CCK-8 assay. Cells (100 μL medium, 2 × 10^5^/mL) were cultured overnight in 96-well plates at 37 °C in humanized atmosphere containing 5% CO_2_, then treated with Fe_3_O_4_@KCTS-LECs-double antibody magnetic nanoprobe (0.1, 0.5, 1.0, 2.0 mg/mL) for 24, or 48 h under the same condition. After incubation, 10 μL CCK-8 solution was added to each well followed for another incubation for 2 h. The optical density (OD) was measured at 450 nm by ELISA microplate reader (Thermo Scientific, USA).

To assess the toxicity of Fe_3_O_4_@KCTS-LECs-double antibody magnetic nanoprobe in vivo, NOD/SCID female mice received a single tail vein injection of 200 μL PBS (control group) or Fe_3_O_4_@KCTS-LECs-double antibody magnetic nanoprobe (2 mg/mL) (*n* = 3 animals per group). After 1 week, mice were sacrificed, sections of major tissues (heart, lung, liver, spleen, kidney) were obtained and immersed in 10% formaldehyde solution, dehydrated, and paraffin-embedded. Paraffinized sections (4 μm thick) were cut and stained with hematoxylin-eosin.

### Date Analysis

SPSS 16.0 statistical software was used for data analysis. The measurement data were expressed as x ± s, the comparison between groups was done by analysis of variance, and the comparison of count data was performed by chi-square test. *P* < 0.05 was considered statistically significant.

## Result

### Principle of a Dual Targeting Magnetic Nanoparticle for Detection of Human Cancer

**Scheme 1:**
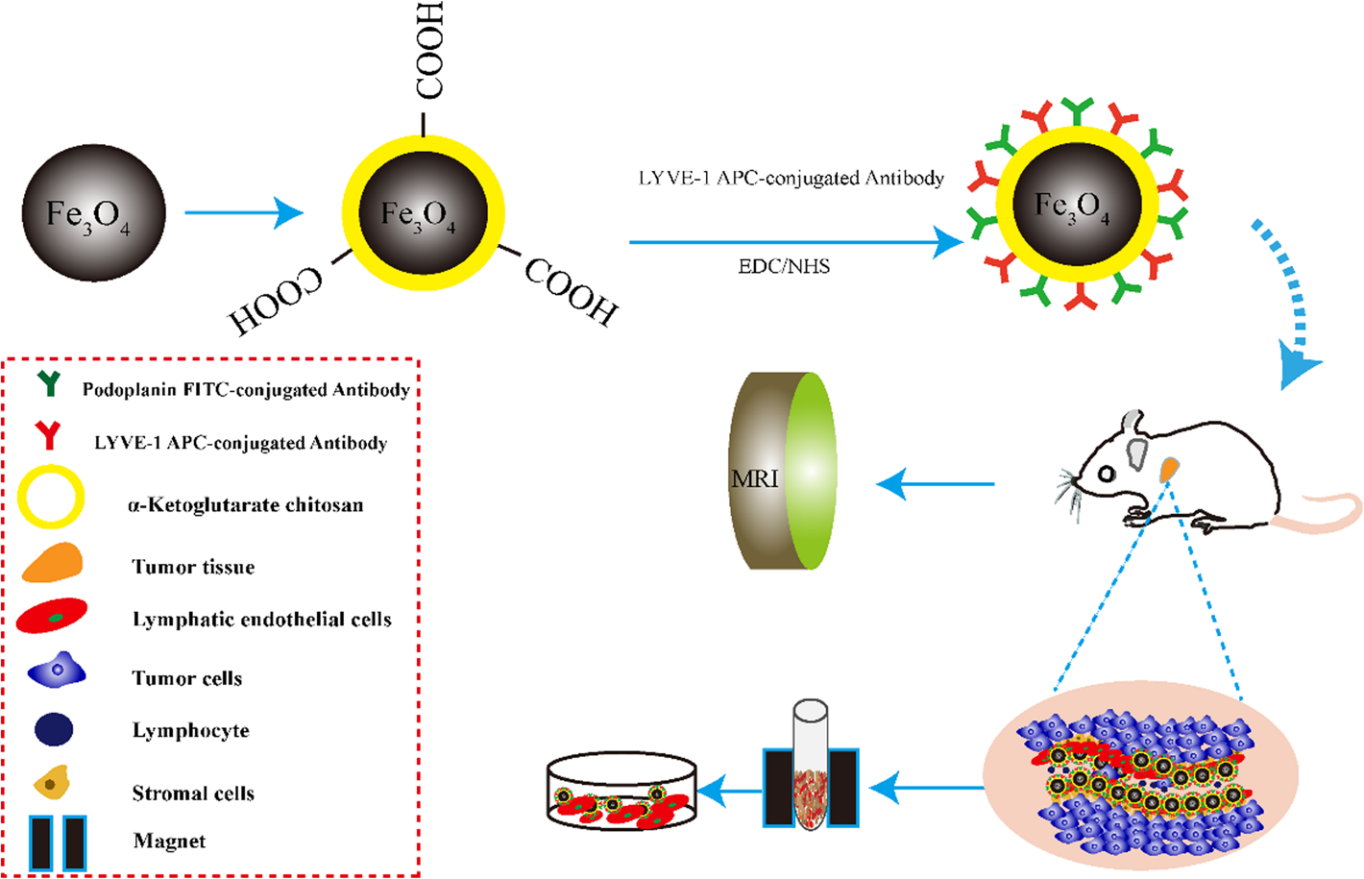
Schematic illustration of Fe_3_O_4_@KCTS-LECs-double antibody magnetic nanoparticles

In this study, as shown in Scheme 1, Fe_3_O_4_@KCTS, a core-shell type of magnetic nanoparticles, was prepared by activating Fe_3_O_4_ with carbodiimide and cross-linking it with α-ketoglutarate chitosan (KCTS). The LYVE-1 and podoplanin antibodies were then added to the surface of these magnetic nanoparticles thereby forming dual-targeting magnetic nanoprobes. These probes were injected into mice models through the tail vein to diagnose the tumor by T2-weighted MR imaging and fluorescence imaging. After excision, the tumor was mashed into a single cell, and then incubated with the probe to identify the lymphocyte endothelial cells with higher purity by a magnet to explore the mechanism of tumor metastasis. The results demonstrated that a dual-targeting magnetic nanoprobe which provides high-purity LECs from tumor tissues was successfully developed, providing a basis for clinical application of LECs in colorectal cancer as well as in early clinical diagnosis by dual-mode imaging of colorectal cancer.

### Characterization of Fe_3_O_4_@KCTS-LECs-Double Antibody Magnetic Nanoparticles

**Fig. 1:**
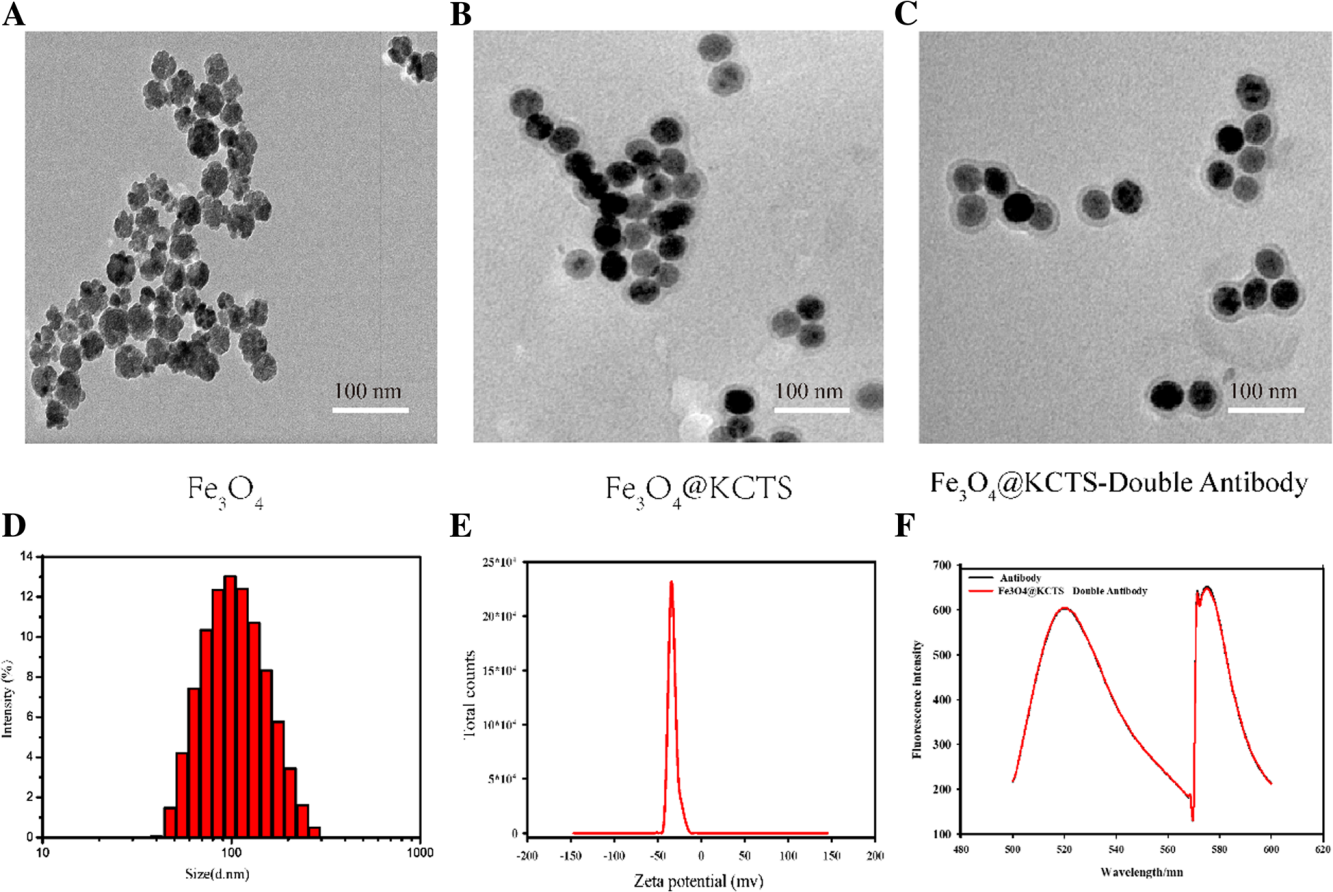
Characterization of magnetic nanoprobe. **a** Transmission electron micrograph image of Fe_3_O_4_ nanoprobe (scale bar, 100 nm). **b** Transmission electron micrograph image of Fe_3_O_4_@KCTS nanoprobe (scale bar, 100 nm). **c** Transmission electron micrograph image of Fe_3_O_4_@KCTS-LECs-double antibody magnetic nanoprobe (scale bar, 100 nm). **d** The particle size was detected by size analysis, and the average size of the probe was 91.28 nm. **e** Zeta potential was about − 32.8 mv. **f** The Fe_3_O_4_@KCTS-LECs-double antibody nanoprobe has emission peaks under excitation light of 488 and 545

TEM images (Fig. 1a) showed that naked Fe_3_O_4_ is irregular with spherical and has agglomerates between the particles. When Fe_3_O_4_ was modified by KCTS (Fig. 1b), the particles were regular spherical or oval, and the particles were uniformly dispersed. As shown in Fig. 1c, Fe_3_O_4_@KCTS-LECs-double antibody magnetic nanoparticles were modified with the negatively charged antibody on the surface of Fe_3_O_4_@KCTS which conferred negative charges, thus increasing the electrostatic repulsion between nanoparticles. Fe_3_O_4_@KCTS-LECs-double antibody magnetic nanoparticles were spherical, uniform, free from particle aggregation, and were not damaged. The average diameter was 91.28 ± 2.31 nm and the PDI was 0.207 ± 0.015 (Fig. 1d). The figure shows that the size distribution of the nanoparticles was narrow and showed good dispersion in water. The negatively charged surface had a zeta potential of − 32.8 ± 1.51 mV, with absolute value of over − 30, indicating that the material had good stability (Fig. 1e).

The results of the fluorescence spectrometer are shown in Fig. 1f. A distinct emission peak was observed in the anti-podoplanin antibody (FITC) under a 488 excitation light, and in the anti-LYVE-1 antibody (APC) under a 545 excitation light. The presence of emission peaks in the Fe_3_O_4_@KCTS-LECs-double antibody complex under 488 and 545 excitation indicates that the materials were coupled successfully.

### Immunohistochemistry and Immunofluorescence

**Fig. 2:**
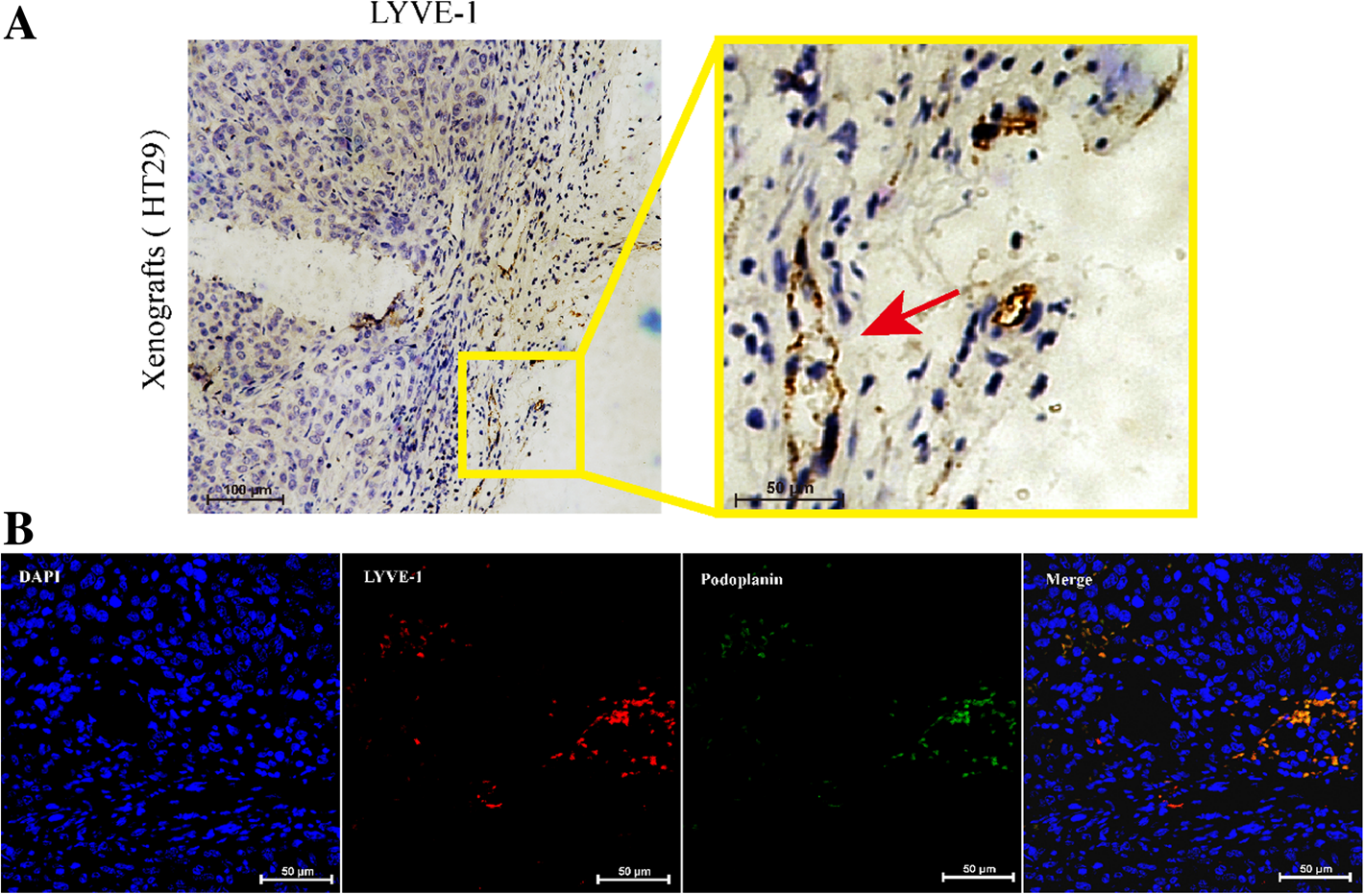
Expression of lymphatic vessels in colorectal cancer tissues. **a** Dilated vessels in the tissue sections were positive for LYVE-1 on endothelial cell membrane by immunohistochemistry (scale bar, 100 μm). On the right is a magnified picture. **b** Dilated vessels in the tissue sections were positive for LYVE-1 and podoplanin on endothelial cell membrane as revealed by immunofluorescence (scale bar, 50 μm)

As shown in Fig. 2a, b, the expression of LYVE-1 in colon cancer tissues and LYVE-1 and podoplanin antibody in frozen tumor tissues was observed. This demonstrated that a small amount of lymphatic endothelial cells were present in colon cancer tissues.

### Isolation and Enrichment of LECs

**Fig. 3:**
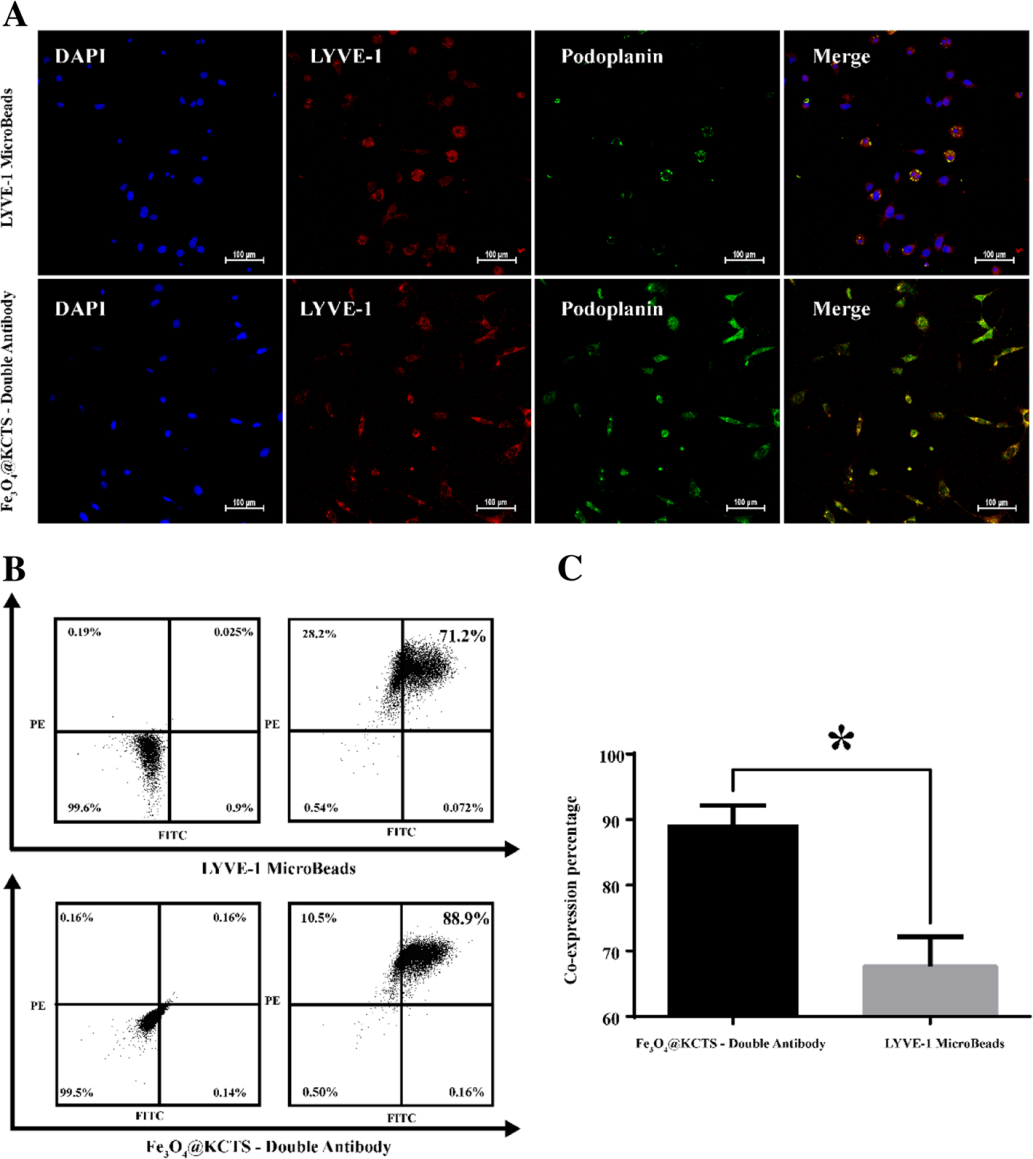
Analysis of the purity of lymphatic endothelial cells. **a** Fluorescence micrographs double-color imaging of LYVE-1 (red) and podoplanin (green)/nuclei [4,6-diamidino-2-phenylindole (DAPI, blue) in the isolated human colorectal cancer LECs sorted using LYVE-1 Microbeads and Fe_3_O_4_@KCTS-LECs-double antibody (scale bar, 100 μm). **b** The co-expression of LYVE-1 (APC) and podoplanin (FITC) as revealed by flow cytometric detection. **c** Results of flow cytometry, *P < 0.05

Immunofluorescence staining was performed on the cells obtained by two different sorting methods. It was found that the cells sorted by LYVE-1 MicroBeads magnetic beads expressed the protein LYVE-1, but not podoplanin, whereas cells obtained by Fe_3_O_4_@KCTS-LECs-double antibody magnetic nanoparticles expressed protein LYVE-1 and protein podoplanin (Fig. 3a). In addition, the flow cytometry results of the cells sorted by different methods were analyzed. As shown in Fig. 3b, the co-expression rate of protein LYVE-1 and protein podoplanin by LYVE-1 MicroBeads magnetic beads was 71.2%, whereas the co-expression rate of the two markers obtained by sorting with Fe_3_O_4_@KCTS-LECs-double antibody magnetic nanoparticles was 88.9%. Two independent sample *t* tests showed that there were statistically significant differences between the two groups (*P* < 0.05) (Fig. 3c).

### Comparison of Biological Functions of LECs Obtained by the Two Different Sorting Methods

**Fig. 4:**
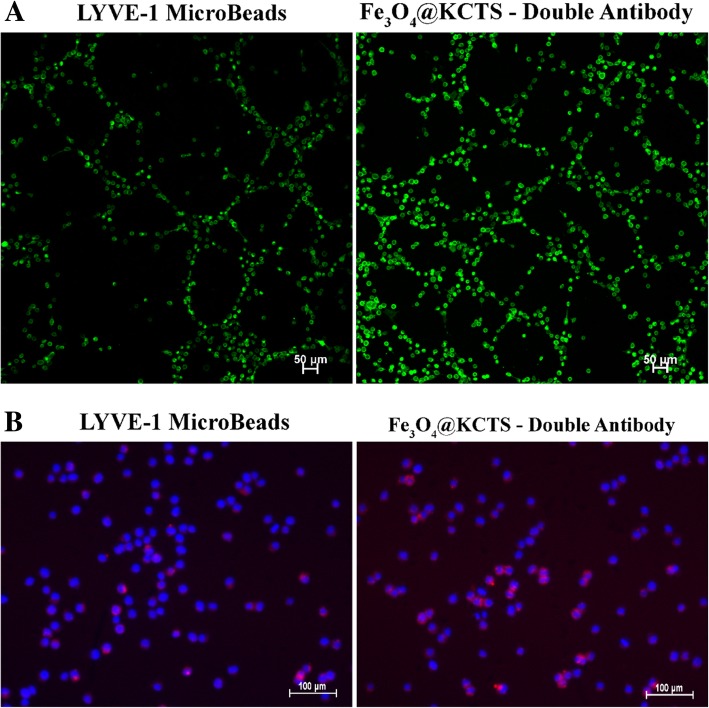
Comparison of biological functions of LECs. **a** Determination of in vitro LECs tube formation ability by Calcein AM (green) in vitro (scale bar, 50 μm). **b** Endothelial cell phagocytosis assay (DiI-labeled, red; DAPI, blue) of endothelial cell function using isolated LECs by LYVE-1 Microbeads and Fe_3_O_4_@KCTS-LECs-Double antibody (scale bar, 100 μm)

We further compared the biological characteristics of lymphatic endothelial cells obtained by two different sorting methods. Matrigel glue tube test results are shown in Fig. 4a. The cells obtained by sorting with Fe_3_O_4_@KCTS-LECs-double antibody magnetic nanoparticles had stronger tube forming ability. The result of Dil-ac-LDL endothelial cell uptake assay shown in Fig. 4b reveals that a red fluorescence was observed in cells sorted by LYVE-1 MicroBeads magnetic beads, but a stronger red fluorescence was observed in the cells sorted by Fe_3_O_4_@KCTS-LECs-double antibody magnetic nanoparticles. These results indicate that the cells obtained by the Fe_3_O_4_@KCTS-LECs-double antibody magnetic nanoparticles had higher purity, better tube forming ability, and endothelial cell phagocytosis, indicating that they are more suitable for research on lymphatic endothelial cells in tumors.

### Bimodal Imaging Analysis In Vivo

**Fig. 5:**
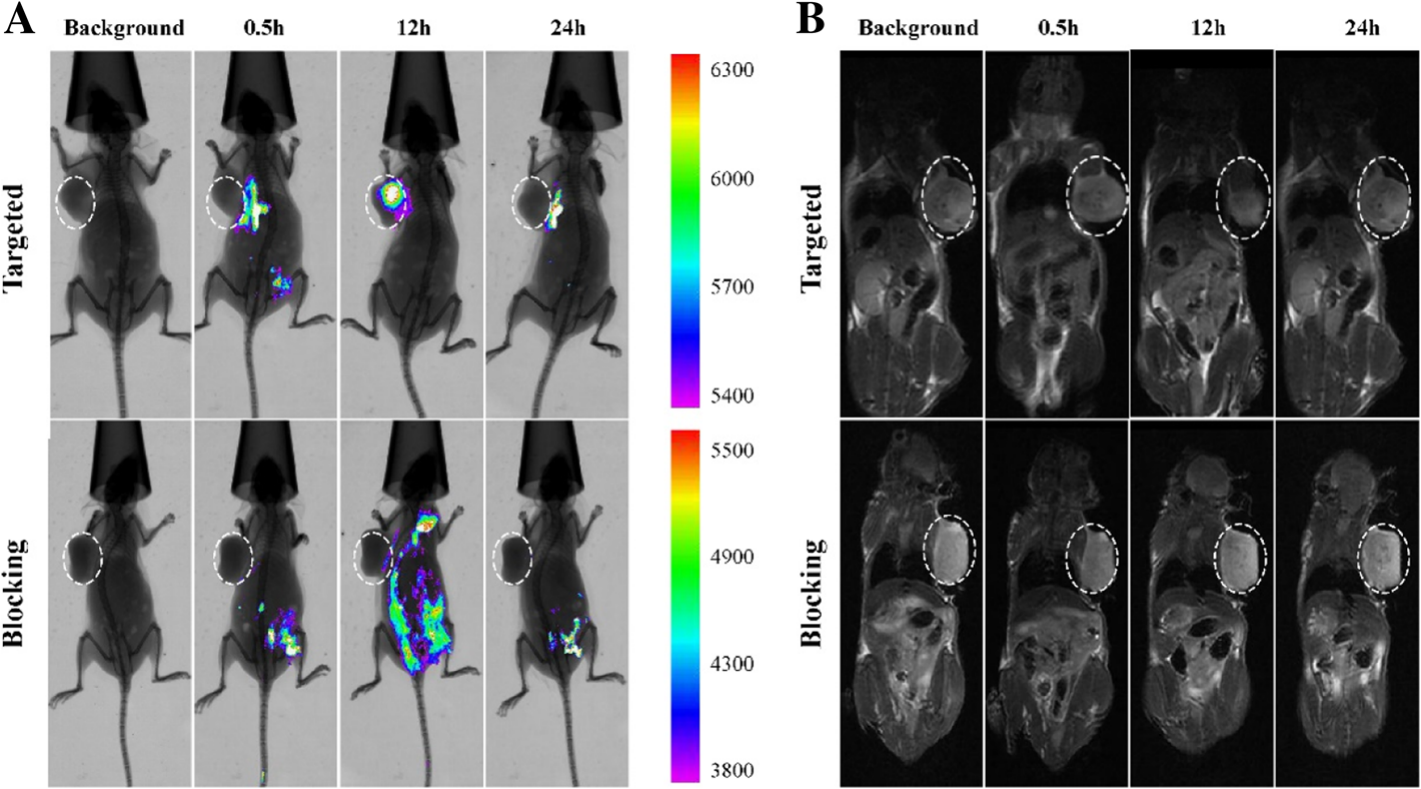
Fluorescence imaging and MRI of colorectal cancer subcutaneous xenografting tumor models of NOD/SCID mice. **a** NOD/SCID mice were anesthetized and imaged with fluorescence imaging system at preinjection and post-injection 0.5 h, 12 h, and 24 h. **b** NOD/SCID mice were anesthetized and imaged with 3.0T MRI scanner at pre injection and post injection 0.5 h, 12 h, and 24 h

The results of in vivo imaging are shown in Fig. 5a. It was found that there was no fluorescence from the tumor before the injection of Fe_3_O_4_@KCTS-LECs-double antibody magnetic nanoparticles. After injection, the fluorescence from the tumor increased, reaching the peak at 12 h. Thereafter, the fluorescence gradually decreased with time. In the control closed group, there was almost no fluorescence from the tumor throughout the experiment. After 24 h, it was observed that the material had been completely metabolized from the mice body of both groups. Similarly, magnetic resonance imaging shown in Fig. 5b revealed that the tumor imaging gradually weakened, reaching its weakest level after 12 h, followed by gradual recovery with time. However, the imaging power of the tumor in the control closed group was almost unchanged. It can be inferred that the Fe_3_O_4_@KCTS-LECs-double antibody magnetic nanoparticles had high tumor targeting properties in vivo.

### In Vitro and in Vivo Toxicity of Magnetic Nanoparticles

**Fig. 6:**
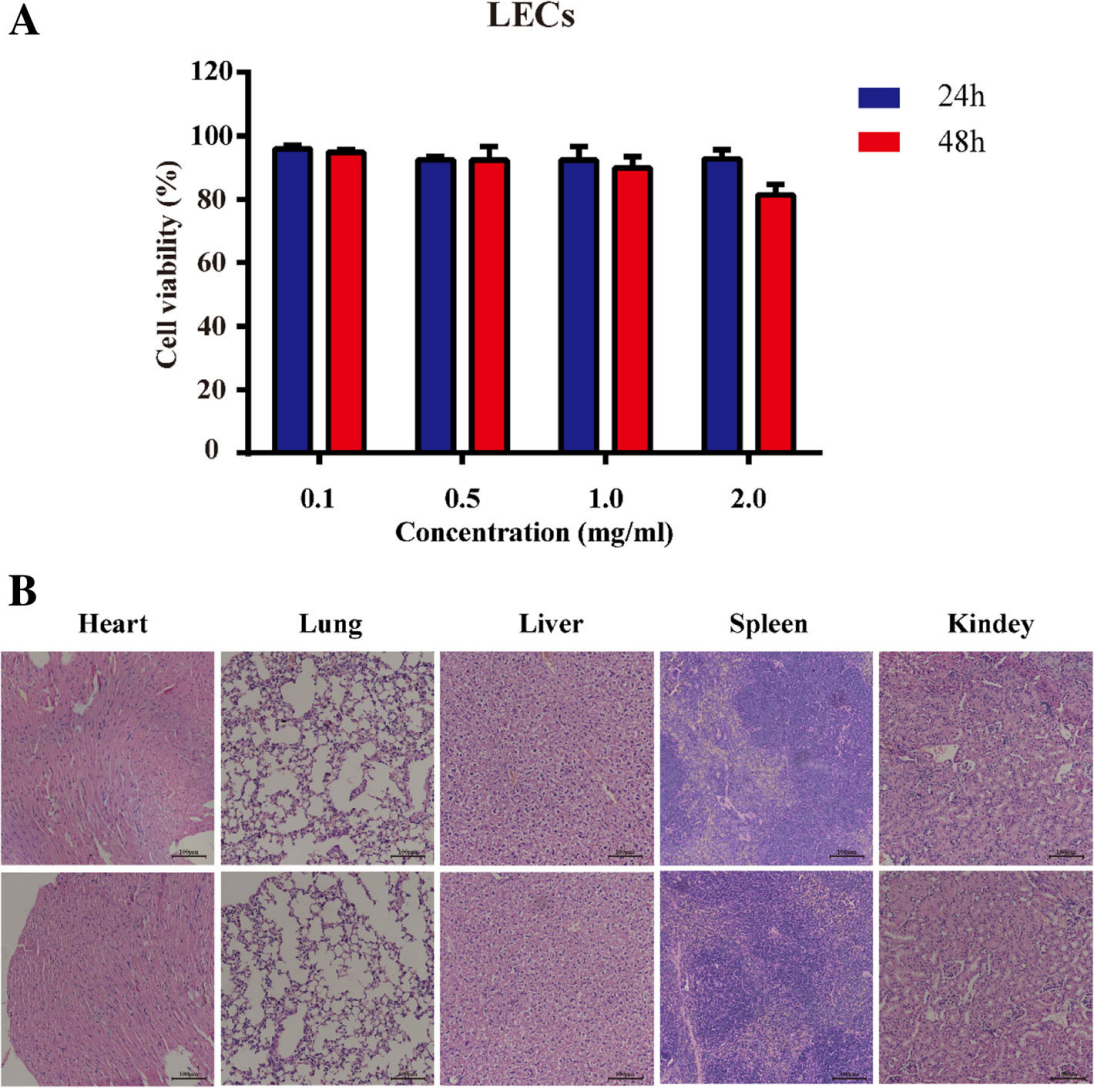
Toxicity of Fe_3_O_4_@KCTS-LECs-double antibody magnetic nanoprobe. **a** LECs were incubated with various concentrations of magnetic nanoprobe, and cell viability was measured at 24 h and 48 h. **b** NOD/SCID mice were treated with PBS or magnetic nanoprobe, and sections from major organs were stained with hematoxylin-eosin and examined by light microscopy (scale bar, 100 μm)

In vitro cytotoxicity of the magnetic nanoprobes was evaluated in the LECs that were sorted by Fe_3_O_4_@KCTS-LECs-double antibody magnetic nanoparticles. The CCK-8 assay results showed that the viability of the cell was high after incubation with various concentrations of magnetic nanoprobe (Fig. 6a). This suggests that Fe_3_O_4_@KCTS-LECs-double antibody magnetic nanoparticles have minimal cytotoxicity. We further evaluated the toxicity of the magnetic nanoparticles in vivo. Female NOD/SCID mice were treated with magnetic nanoprobe and then tissue sections from major organs after staining with hematoxylin-eosin were examined. As shown in Fig. 6b, no obvious signs of necrosis or inflammation were observed. These results suggested that the magnetic nanoprobe did not produce toxic effects in vivo. These findings indicate that the magnetic nanoprobe designed in this study is suitable for application as a detection probe in animal and human investigations.

## Discussion

It has long been know that transportation through the lymphatic system is an important pathway for tumor cell proliferation. However, compared with vascular endothelial cells (BECs) in blood vessels, LECs, as major components of lymphatic vessels, have not been fully studied. Identification of LECs markers will promote investigations on LECs and make it easier to distinguish them from BECs. LYVE-1 is a type I intact transmembrane glycoprotein receptor composed of 322 amino acid residues [20, 21], and it is presently considered to be the most specific lymphatic endothelial cells marker [22, 23]. Podoplanin is a type I transmembrane salivary mucin-like glycoprotein with a molecular weight of 38 kDa. Current research shows that podoplanin can distinguish lymphatic vessels and blood vessels very well [24, 25]. Therefore, it can be combined with other lymphatic markers to identify LECs. In this study, two antibodies were used to sort LECs, and the purity of the sorted cells was verified by immunofluorescence co-localization, flow double staining, and co-expression. The cells sorted by Fe_3_O_4_@KCTS-LECs-double antibody magnetic nanoparticles displayed similar biological characteristics with LECs, and had stronger tube-forming ability and better phagocytic ability.

Recently, immunomagnetic-based purification methods have been used to separate different cells from mixed cultures, while magnetic beads coated with LYVE-1 or podoplanin antibodies have been used to separate specific subpopulations from crude cells populations [10, 26]. These methods only purify the target subgroups from mixed cultures. It is likely that this not only leads to incomplete isolation of LECs, but also causes contamination of heterogeneous cells such as vascular endothelial cells. Furthermore, these methods are time-consuming and are costly. Previously, several specific lymphoid markers were reported, e.g., LECs were isolated from different tissues primarily by the means of collagenase treatment. However, during primary culture, collagenase treatment may produce a mixture of cells, including vascular endothelial cells and interstitial cells.

In this study, the Fe_3_O_4_@KCTS-LECs-diabody magnetic nano-double targeting probe was constructed using chitosan-coated ferroferric oxide which binds to highly specific target molecules LYVE-1 and podoplanin expressed on lymphatic endothelial cells. The electron microscope analysis showed that the probe had uniform appearance and good dispersibility. Zeta potential reflects the stability of a molecular probe. Highly positive or negative zeta potential values reflect high stability and low capacity to aggregate. It is generally believed that molecules with zeta potential greater than + 30 mV or less than − 30 mV have good stability. In this experiment, the probe had a zeta potential of − 32.8 ± 1.51 mV, which indicates that the probe had good stability.

Fe_3_O_4_ is a magnetic nanoparticle oxide which has been widely used in modern medicine and biology because of its unique physical properties. It is particularly used in diagnosis systems based on magnetic resonance imaging and magnetic hyperthermia as well as in cell separation applications. The contrast agents used in MR imaging can be divided into paramagnetic iron oxide nanoparticles (T2-negative contrast agent) and Gd compounds (T1-positive contrast agent). Fe_3_O_4_ nanoparticle resonance contrast agent is considered to be a R2-weighted, and its performance is often affected by some factors, such as (a) composition, (b) NP size, (c) surface coating, and (d) synergistic magnetic effect produced by multiple superparamagnetic Fe_3_O_4_ NP centers in a small volume [27]. In addition, it can be used in sorting of target cells under the magnetic field effect, that is, after the target cells are combined with specific probes, the target cells are sorted by magnet adsorption. For example, the lymphatic endothelial cells sorted using the probe designed in this study had better tube forming ability and endothelial cell phagocytosis. The relaxation parameter R1 or R2 is typically used to measure the quality of the MRI contrast agent, which describes the ability of the contrast agent T1 or T2 relaxation time [28]. The current NP-based T1 contrast agents are associated with cell toxicity, generating the need for better alternatives. Fe_3_O_4_ provides good biocompatibility compared to the above-described cerium-based materials [29]. In this study, we studied the in vitro and in vivo toxicity of the nano-probes modified by chitosan on the surface of Fe_3_O_4_. Our results showed that the probe was suitable for sorting of specific cells, and had low toxicity. It is therefore an ideal material that can be used for culturing of the sorted cells and further research on tumor metastasis through lymphatic vessels.

Here, specific target molecules such as peptides, antibodies, aptamers, etc. were attached to the surface of magnetic nanomaterials, thereby facilitating tumor diagnosis and treatment [30, 31]. This study successfully constructed the Fe_3_O_4_@KCTS-LECs-double antibody magnetic nano-double targeting probe. This probe could bind to specific target molecules, and hence can be used to separate lymphatic endothelial cells and MR/fluorescence imaging.

## Conclusions

In summary, a dual targeting magnetic nanoparticle that can detect human cancer is presented in this study. The magnetic nanoparticle probe displays high biosafety and stability. The probe could bind to specific target molecules, thereby enabling sorting of lymphatic endothelial cells. The double antibody coating customized by incorporating self-made magnetic beads produced enabled separation of pure LECs, which reduced sorting time, improved cell activity, and reduced cost. These findings indicate that the magnetic nanoprobe designed in this study is suitable for application as a detection probe in animal and human investigations. This study provides a platform for studying the mechanisms of lymphatic metastasis and role of lymphatic endothelial cells in tumors. Moreover, the Fe_3_O_4_@KCTS-LECs-double antibody magnetic nanoparticles can be applied in fluorescence/MR molecular imaging in vivo, enabling clinical diagnosis of cancer.

## Abbreviations

DAPI: 4′,6-Diamidino-2-phenylindole
DMEM: Dulbecco’s modified Eagle medium
EDC: 1-Ethyl-3-(3-dimethylaminopropyl) carbodiimide hydrochloride
FBS: Containing no fetal bovine serum
Fe_3_O_4_: Magnetic ferroferric oxide
HT29: Human colon cancer cell
KCT: α-Ketoglutarate carboxymethyl chitosan
LECs: Lymphatic endothelial cells
LYVE-1: Lymphatic vessel endothelial hyaluronan receptor 1
MR: Magnetic resonance
NHS: N-hydroxysuccinimide
T2: Transverse (spin-spin) relaxation time
TEM: Transmission electron microscopy
VEGFR-3: Vascular endothelial growth factor receptor 3
